# 

**Published:** 1904-12-15

**Authors:** 


### ANNOUNCEMENTS.

THE *Dental Surgeon*, is the title of a new dental journal published every week by Bailliere, Tindall & Cox, No. 8 Henrietta Street, Covent Garden, London Eng., and is edited by Robert Manning, L. D. S. The object sought in the editor's "Foreword" is to take away the dentist's excuse for not reading. Which he interprets to be, that there is is nothing worth reading. It is to be an independent but courteous journal, and will print the news fresh every week, and expects to succeed. If it realizes its aims, it certainly will succeed and we wish it may do so.

P. BLAKISTON'S SON & CO., have just issued their physician's visiting list for 1905. It is the 54th year of publication and is the most popular book of its kind ever published. It contains a complete day book and abbreviated system of keeping accounts which is all that can be desired.

THE Institute of Dental Pedagogics will hold its next meeting at Louisville, Ky., December 26th to 30th. This is one of the best dental meetings of the year and every teacher and practitioner who is interested in educational efforts, should certainly attend this meeting. The programs are always full of timely papers and earnest discussions. We regret we haven't the program for announcement.

